# Postoperative recurrence of mixed extragonadal germ cell tumor in the right shoulder: a case report

**DOI:** 10.1186/s13000-023-01312-0

**Published:** 2023-02-20

**Authors:** Yao Li, Xiang Huang, Xue Meng, Yuqing Luo, Shuai Luo, Jinjing Wang

**Affiliations:** Department of Pathology, Affiliated Hospital of Zunyi Medical University, Zunyi City, Guizhou Province P.R. China

**Keywords:** Germ cell tumor, Extragonadal, Yolk sac tumor, Immature teratomas

## Abstract

**Background:**

Extragonadal germ cell tumours (EGGCTs) originated in Shoulder are extremely rare, with 1 case described in the literature. We report a case of a patient with a primary Right Shoulder mixed EGGCT.

**Case presentation:**

A 36-year-old male patient was hospitalized for 6 months due to progressive right shoulder swelling accompanied by pain. Subsequently, the right shoulder tumor was removed entirely. Gross pathological examination showed that the size of the tumor mass was about 14 × 10 × 6 cm.Mutations were observed in ENPEP (4q25), ZCCHC11, RREB1 (6p24.3), CKAP4 (12q23.3), and other genes were detected by whole exome sequencing. Histology revealed a mixed EGGCT of the Right Shoulder with immature teratoma and yolk sac tumour. The patient went through 6 cycles of chemotherapy. After 7 months of follow-up, the patient is recurrence.

**Conclusion:**

The primary MEGCT of the shoulder is an extremely rare condition. However, the recurrence and metastasis rates are high. Therefore, further research is necessary to determine this rare disease’s genetic and clinical characteristics to develop an effective treatment plan.

## Background

Germ cell tumors can be divided into two types: seminoma and non-seminoma. The non-seminoma tumors can be further classified as embryonal carcinoma, yolk sac tumor, chorionic cell carcinoma, mixed germ cell tumor (MGCT), and teratoma. MGCT consists of two or more germ cell components. And mainly occurs in the gonads (testes and ovaries) and is rarely found in the extragonadal organs called Extragonadal germ cell tumors (EGCT). EGCT often occurs in the midline of the body. The most common sites reported in adults are the anterior mediastinum, retroperitoneum, pineal gland, and suprasellar area. They can also be found in the prostate gland, bladder, stomach, ear and orbit, liver, omentum, esophagus, rectum, and rarely in the pericardium. To the best of our knowledge, there has been only one reported case of mixed extradadal germinoma in the right shoulder [1].

## Case presentation

A 36-year-old male patient was hospitalized for 6 months due to progressive right shoulder swelling accompanied by pain. The patient was previously in good health. Physical examination showed that the right shoulder joint had a hard lump behind it, accompanied by obvious tenderness, no ecchymosis on the skin, low skin temperature, and no other related symptoms.

**Fig. 1:**
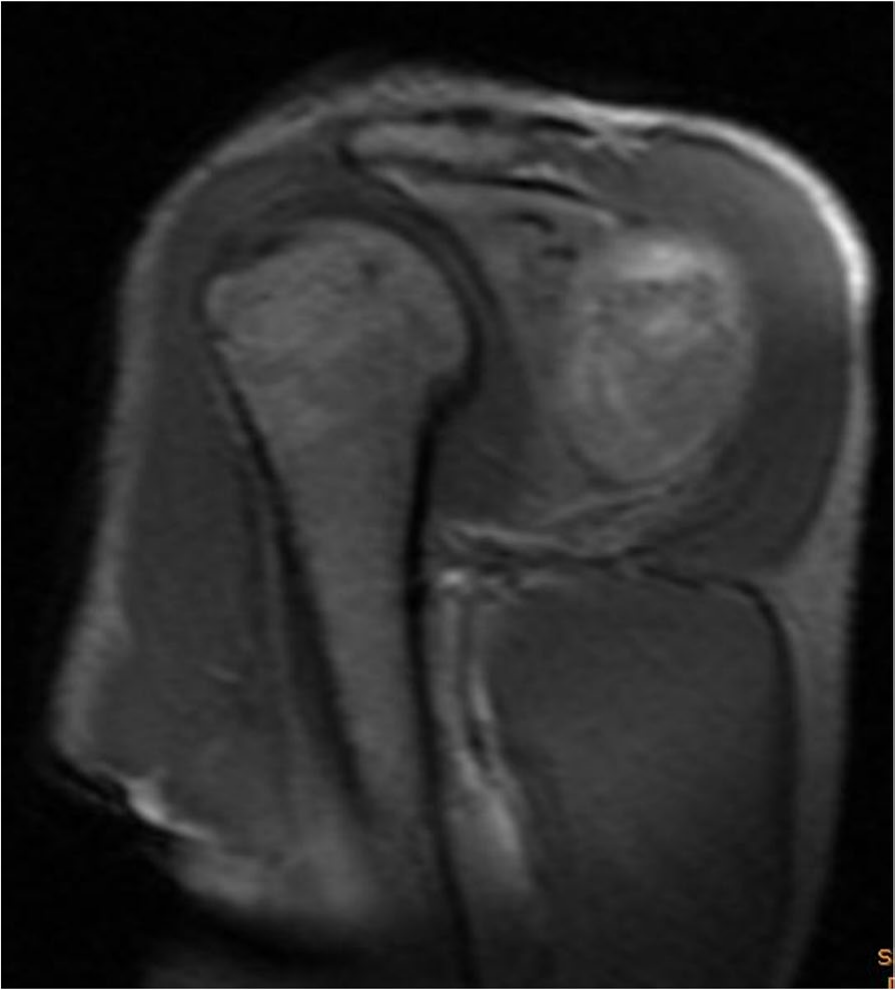
MRI of the right shoulder shows that tumor mass was 13.2 × 4.1 × 7.8cm in size, uneven long T2 and long T1 signal mass behind the right shoulder joint and deep in the deltoid muscle

Magnetic resonance imaging (MRI) showed a mass located in the posterior part of the right shoulder joint and the deep part of the deltoid muscle measuring 13.2 × 4.1 × 7.8 cm with uneven long T2 and long T1 signals (Fig. 1). The mass had a clear boundary and obvious enhancement on the edge of the enhanced scan and was considered to be a malignant tumor. Ultrasound examination of the reproductive system showed no abnormalities.

**Fig. 2:**
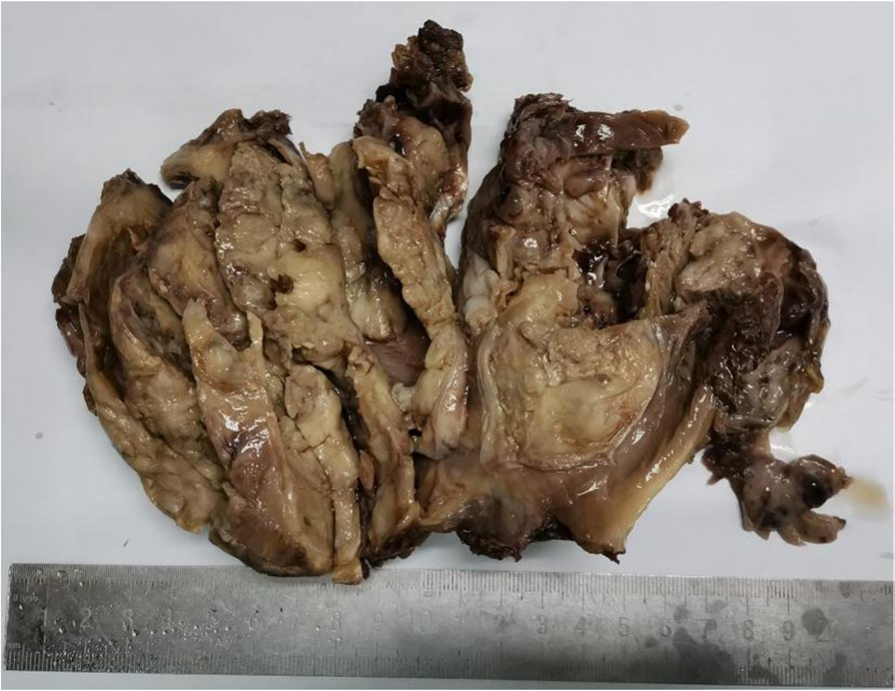
Macroscopic aspect the color of the solid area of the mass was gray white and gray yellow

Subsequently, the right shoulder tumor was removed entirely. Gross pathological examination showed that the size of the tumor mass was about 14 × 10 × 6 cm. The boundary between the mass and the surrounding tissue was unclear; no capsule and the section had two components. Biopsy was conducted on the tumor mass, and the results revealed the color of the solid area of the mass was gray white and gray yellow (Fig. 2), medium in quality, and had a fish-like appearance. The cystic area contained dark red liquid. Postoperative histopathological diagnosis was MGCT in the right shoulder.

After the surgery, the patient received six courses of chemotherapy. However, 7 months later, the patient complained of chest pain and was hospitalized again. MRI was again performed on the patient. The MRI results showed cystic and solid mixed abnormal signals in the medial soft tissue of the right shoulder and the upper segment of the right humerus, with uneven enhancement, predicted to be the recurrence of MGCT. Chest enhanced CT showed a round-like mass in the upper lobe of the right lung near the hilum, suggestive of MGCT metastasis to the lung. Resection of the right scapular mass and surrounding tumor tissue was performed, and the postoperative pathology confirmed the recurrence of MGCT.

**Fig. 3:**
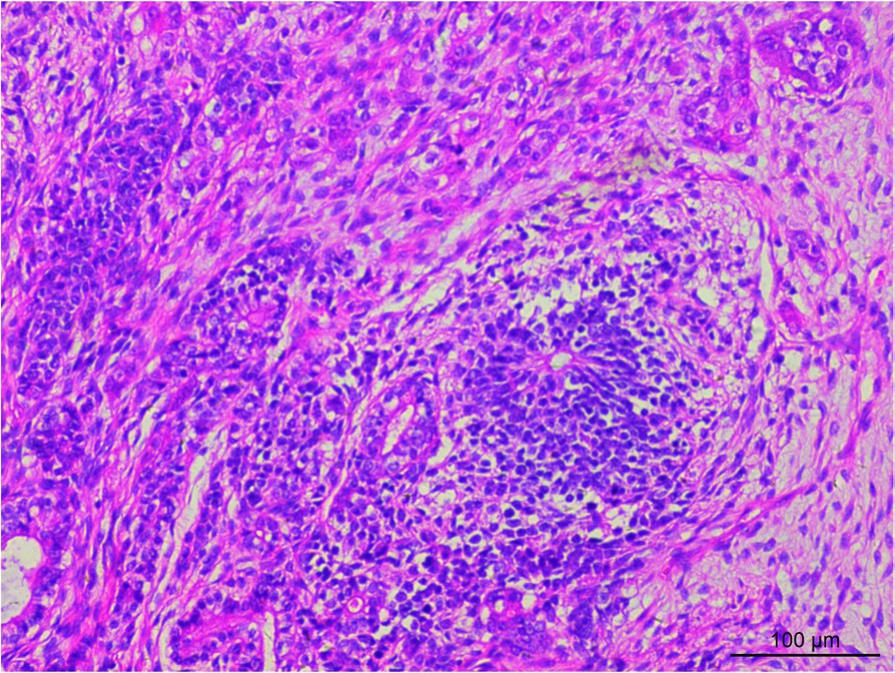
H&E staining visualized at 200 × magnification primitive neural tube, and daisy-shaped mass were found in tumor tissue

**Fig. 4:**
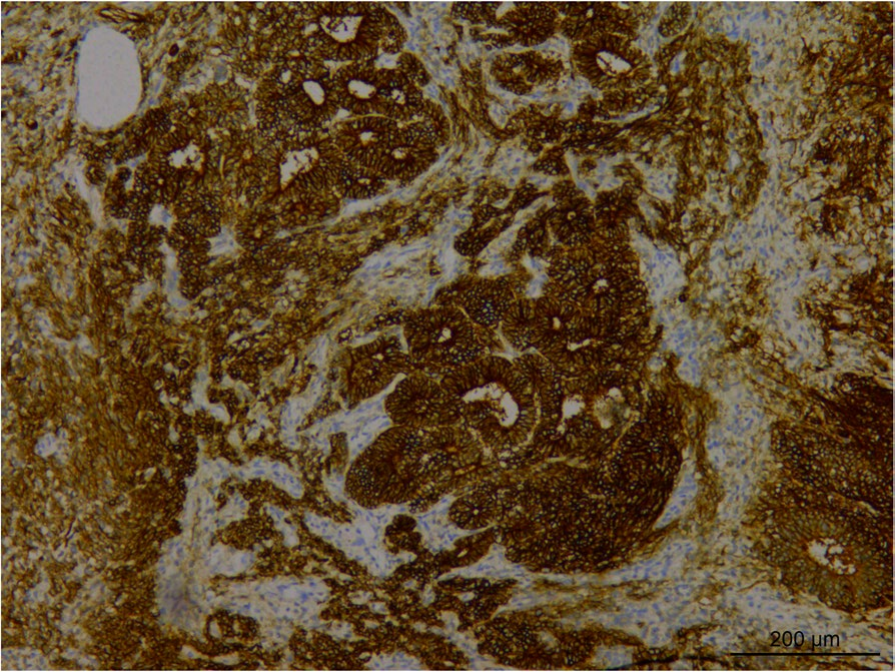
Immunohistochemical staining visualized at 100 × magnification. The immature nerve tissue was positive for CD56 expression

**Fig. 5:**
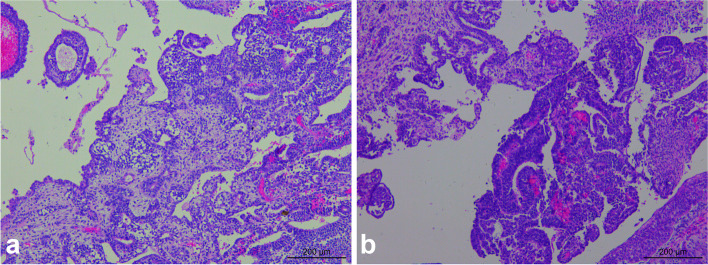
H&E staining visualized at 100 × magnification. The tumor tissue is irregular and acinar like structure, lined with single-layer flat or cubic cells (**a**), papillary structures can be observed (**b**)

**Fig. 6:**
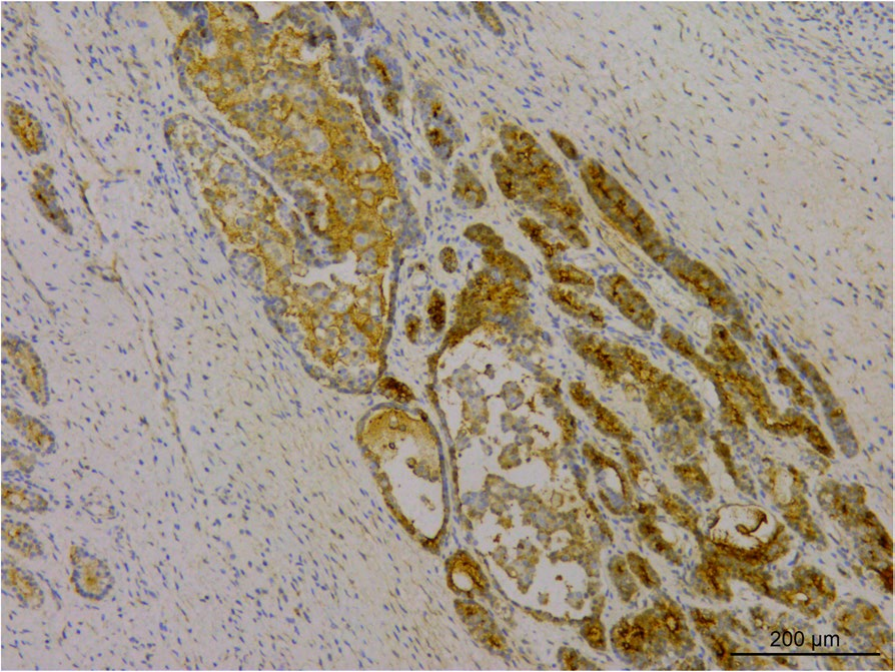
Immunohistochemical staining visualized at 100 × magnification. The expression of Glypican-3 was positive in the yolk sac tumor component of the tumor

**Fig. 7:**
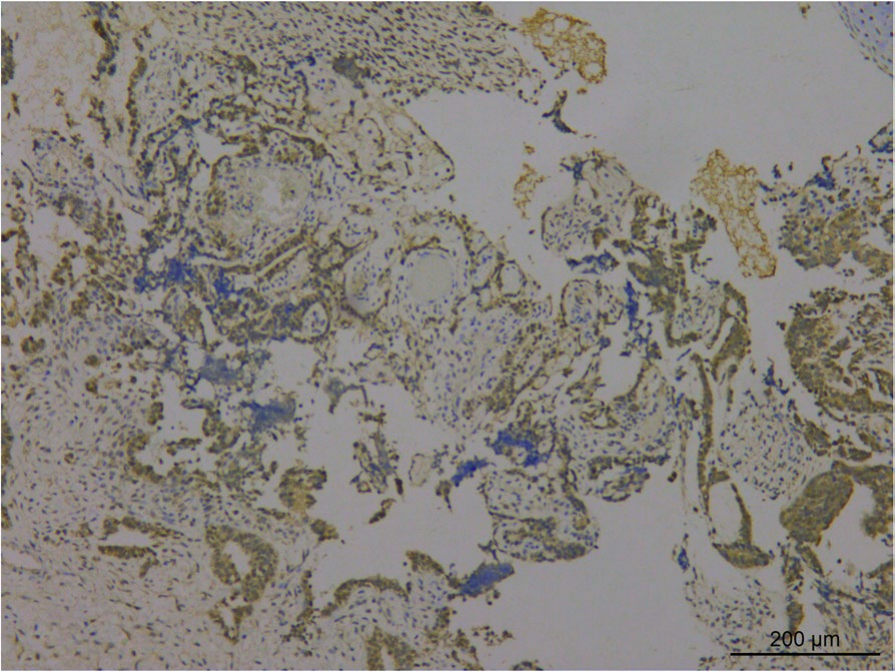
Immunohistochemical staining visualized at 100 × magnification. The expression of AFP was positive in the yolk sac tumor component of the tumor

The sections were stained using H & E staining to study the morphology of the tumor. Histological analysis revealed that the tumor tissue had diverse morphology, and the lesions were mainly of immature teratoma. The tumor mass was mainly composed of the cartilage of embryonic origin, which was diffusely distributed in immature nerve tissue. Primitive neural tubes and daisy-shaped clusters were found in the neural component of tumor tissue (Fig. 3), with overlapping, over-stained cells and many nuclear divisions were observed in the tumor. The tumor cells stained positive for CD56 (Fig. 4), CD99, and S-100, and negative expression of Glypican-3, NSE, SYN, and CGA was observed using immunohistochemical analysis. Further, the second tumor detected was the yolk sac tumor. The tumor tissue is irregular and acinar like structure, lined with single-layer flat or cubic cells (Fig. 5a), papillary structures can be observed (Fig. 5b). Immunohistochemical analysis of the tumor tissue was positive for Glypican-3 and AFP expression (Figs. 6 and 7). Additionally, positive expression of SALL4, PLAP, and CK was observed in both the tumor regions, while OCT4, HCG, and CD117 expression were negative. Ki-67 accounted for about 80% of staining. Mutations were observed in *ENPEP* (4q25), *ZCCHC11*, *RREB1* (6p24.3), *CKAP4* (12q23.3), and other genes were detected by whole exome sequencing.

To conclude, the histopathological and immunohistochemical analysis revealed MGCT on the patient’s right shoulder was composed of two types of tumors, of which 70% was made of immature teratoma, which was classified as grade III and yolk sac tumor accounted for the remaining 30%.

## Discussion

Germ cell tumors usually occur in the gonads, while only 2- 5% of germ cell tumors occur outside the gonads [2]. The diagnosis of germ cell tumors in extragonadal organs should exclude the possibility of their metastasis from primary germ cell tumors of the testis and ovaries. It is important to note that often EGCT is considered a result of metastases from the occult adenocarcinoma. Therefore, imaging of the gonads should rule out gonadal tumors while diagnosing EGCT. In our patient’s case, the imaging of the gonads revealed the patient’s testes were normal.

Gonadal germ cell tumors originate from germ cells (primordial germ cells) at different stages of differentiation. It is believed that during the differentiation process, the primordial germ cells do not migrate to gonads and instead are distributed to other tissues and organs, resulting in abnormal hematopoietic or immune function, thereby leading to EGCT. Interestingly the morphology of EGCT is identical to the germ cell tumors originating from gonads. However, this hypothesis needs further validation.

MGCT are rare and composed of two or more germ cell components, of which at least one of the germ cell components remains the same as the original component [3]. The most common type of MGCT is the mixture of teratoma and embryonic carcinoma, and other tumor components include yolk sac tumor, seminoma, and choriocarcinoma. Our patient’s tumor mass included immature teratoma and yolk sac tumor components. MGCT has a diverse histomorphology, so determining the tumor’s origin is difficult. Hence immune markers play an important role in determining the source of tumors [4]. Teratomas are the most common germ cell tumor, which can be further classified into mature and immature teratomas. Mature teratomas are mainly composed of mature blastoderm components and have higher incidences than immature teratomas. In our patient’s case, the tumor tissue was composed of scattered cartilage and adenoid structures, consistent with the morphology of mature teratoma. Further, immature teratomas have various immature components from three germ layers and neural tissues, such as neuroepithelial daisy clusters and neural tube-like structures. Immature mesenchymal tissues, such as cartilage, bone-like fat, and striated muscle tissue, are also seen. In this patient’s case, the tissue mass had primitive neural tubes and daisy-like structures and was positive for the immunophenotypes SALL4, CD56, and CD99, confirming the diagnosis of immature teratoma. Immature teratomas are histologically graded according to the area of the immature neuroepithelium. Less than 1/40HPF is classified as grade I, benign, with malignant potential; (1 ~ 3)/40HPF is grade II, low-grade malignancy; > 3/40HPF is classified as malignant grade III. Biopsy revealed that the area of immature neuroepithelium was more than three 40 times the visual field; hence, this patient’s tumor was confirmed to be grade III.

Yolk sac tumors have a variety of shapes like loose myxoid matrix, sieve-like microcapsules, labyrinth-like fissures, characteristic reticular, solid, glandular duct acinar, endodermal sinus, papillary, and multivesicular yolk sac-like structure, etc.; Characteristic Schiller Duval corpuscles and hyaline corpuscles are often seen in the tissue of yolk sac tumor. The characteristic marker of yolk sac tumor is AFP. However, immature teratomas express AFP, so that they can be nonspecific. Therefore, Glypican-3 and AFP were used to check for the presence of yolk sac tumors in the patient’s biopsy [5].

Embryonal carcinoma is composed of large and primitive epithelioid cells arranged in patches or irregular adenoids, fissures, tubes, or papillae and expressed CD30. In our patient’s case, the expression of Glypican-3 and AFP was positive, whereas CD30 and HCG expression was negative. These results ruled out the possibility of embryonic and choriocarcinoma and confirmed the second component of the tumor mass was the yolk sac tumor.

It has been reported that EGCT and gonadal GCT have the same cytogenetic characteristics. Fluorescence in situ hybridization has demonstrated that amplififi cation on chromosome 12p, particularly 12p13, occurrs in nearly all Intracranial germ cell tumors (IGCTs), regardless of histological subtypes。A further study findings in the molecular characterization of IGCTs suggest roles of CCND2, RB1, and PRDM14 in the pathogenesis of IGCTs and identify the KIT/RAS and AKT1/mTOR pathways as potential therapeutic targets in future [6]. However, whole exome sequencing was performed using the patient’s DNA, and the results reveal that the patient had mutations in *ENPEP* (4q25), *ZCCHC11, RREB1* (6p24.3), *SKAP4* (12q23.3), and other genes. *ENPEP* encodes for glutamyl aminopeptidase, which catalyzes the cleavage of the N-terminal aspartic acid from angiotensin II. It is associated with tumorigenesis and immune microenvironment and can predict the efficacy of immune checkpoint inhibitors [7]. *ZCCHC11* is an RNA binding protein involved in the post-transcriptional regulation of various RNAs. It alters the cell cycle by promoting the transition from G1 to the S phase, which affects cell proliferation [8]. Responsive element binding protein 1 (*RREB1)* is a protein-coding gene associated with chromosome 22q11.2 deletion syndrome (22qDS), which may be related to MGCT [9]. Cytoskeleton-associated protein 4 (CKAP4) binds to the DKK-1 and p85α subunit of PI3K to activate PI3K/AKT signal pathway to stimulate cancer cell proliferation [10]. These results may aid in enhancing our understanding of the pathogenesis and treatment of germ cell tumors. However, additional studies are needed to verify this data further.

Reports suggest MGCT with malignant components (other germ cell tumors, cancers, or sarcomas) are the most aggressive in nature. Two years follow-ups reveal that the survival rate is less than 50% due to local invasion or distant metastasis (lymph nodes, liver, lung, heart, bone, and brain) [11]. Owing to the high degree of malignancy, MGCT treatment involves surgery, supplemented by chemotherapy and radiotherapy. Low-dose radiotherapy combined with chemotherapy can reduce mortality and improves the prognosis. Our patient received chemotherapy alone post-surgery, and the tumor relapsed after 7 months of treatment; this could be attributed to the lack of radiotherapy, localized invasion, and metastasis of MGCT [12], coupled with poor prognosis.

## Conclusions

In conclusion, the primary MEGCT of the shoulder is an extremely rare condition. The site of MEGCT are often hidden, accompanied by non-specific symptoms, and can be easily missed diagnosed or mistaken for other tumors. Pathological examination and diagnosis are essential for the treatment and prognosis of patients. Hence procurement of the whole tumor sample and extensive histological analysis is a prerequisite for effective treatment. Early diagnosis and timely chemotherapy can prolong the survival time of patients. Surgery is currently the primary treatment, followed by chemotherapy and radiotherapy. However, the recurrence and metastasis rates are high. Therefore, further research is necessary to determine this rare disease’s genetic and clinical characteristics to develop an effective treatment plan.

## Data Availability

All the data regarding the findings are available within the manuscript.
